# Lifestyle intervention in individuals with impaired glucose regulation affects Caveolin-1 expression and DNA methylation

**DOI:** 10.1080/21623945.2020.1732513

**Published:** 2020-03-03

**Authors:** Helene A. Fachim, Kirk Siddals, Nagaraj Malipatil, Rachelle P Donn, Gabriela YC Moreno, Caroline F Dalton, Safwaan Adam, Handrean Soran, J Martin Gibson, Adrian H Heald

**Affiliations:** a Faculty of Biology, Medicine and Health and Manchester Academic Health Sciences Centre, University of Manchester, Manchester, UK; b Department of Diabetes and Endocrinology, Salford Royal NHS Foundation Trust, Salford, UK; c Dirección General de Calidad y Educación en Salud, Secretaría de Salud, Mexico City, Mexico; d Biomolecular Science Research Centre, Sheffield Hallam University, Sheffield, UK; e Department of Endocrinology, The Christie NHS Foundation Trust, Manchester, UK; f Department of Endocrinology, Diabetes and Metabolism, Manchester Royal Infirmary, Manchester, UK; g Lipoprotein Research Group, Division of Cardiovascular Sciences, School of Medical Sciences, Faculty of Biology, Medicine & Health, University of Manchester, Core Technology Facility, Manchester, UK

**Keywords:** Impaired glucose regulation, DNA methylation, bariatric surgery, Caveolin 1

## Abstract

Aims: We investigated whether a lifestyle intervention could influence expression and DNA methylation of diabetes-related genes in patients with impaired glucose regulation (IGR), the results were compared to bariatric surgery, considering it an intensive change. Methods: Twenty participants with IGR had adipose tissue biopsy and blood collected pre- and post-lifestyle (6 months) intervention; 12 obese patients had subcutaneous fat taken before and after bariatric surgery. RNA/DNA was extracted from all samples and underwent qPCR. DNA was bisulphite converted and 12 CpG sites of Caveolin-1 (*CAV1*) promoter were pyrosequenced. Results: lifestyle intervention resulted in opposite direction changes in fat tissue and blood for *CAV1* expression and DNA methylation and these changes were correlated between tissues, while no significative differences were found in *CAV1* expression after bariatric surgery. Conclusions: Our findings suggest a role for *CAV1* in modulating adipocyte function as a consequence of lifestyle changes, as exercises and diet. These results may provide insights into new therapeutic targets for diabetes prevention.

## 1. Introduction

Type 2 diabetes mellitus (T2DM) in parallel with obesity is responsible for a major reduction in life expectancy and quality of life, increasing health-care costs [1]. Prediabetes or impaired glucose regulation (IGR) is a condition that precedes T2DM and can be identified by mildly elevated glycated haemoglobin A1 c (HbA1 c) [2,3]. The mechanisms which underlie the evolution from IGR to T2DM are not currently understood.

In order to prevent patients with IGR becoming diabetic, hospitals around the world implement programmes to reduce the risk of IGR progression [2]. Without any lifestyle or medical intervention, in particular weight loss and physical activity, more than 50% of people who have IGR will develop T2DM, accompanied by an increased risk of cardiovascular disease and cardiovascular death over a period of 10 y [4,5].

In Salford, UK we have a lifestyle change programme called Care Call (http://www.srft.nhs.uk/EasysiteWeb/getresource.axd?AssetID=119162&type=full&servicetype=Inline) [6]. This is a non-face-to-face telephone-based modular intervention programme, which has been proven to benefit people with impaired glucose regulation [7].

Both genetic and environmental factors may contribute to the progression from IGR to T2DM, as may a variety of other mechanisms that may mediate the effect of drugs or other environmental influences on clinical outcomes. One well-established process, that might be particularly likely to interact with other genetic factors, is that of epigenetics. Epigenetic mechanisms can be defined as changes of a phenotype such as the gene expression of a specific cell type that are not caused by changes in the nucleotide sequence of the genetic code itself [8]. Two such mechanisms are DNA methylation and histone modifications; both affect gene expression by changing the profiles of proteins which bind to specific DNA regions, such as transcription factors into promoter gene sequences [9]. As these DNA methylation marks can be dynamically regulated and programmed by environmental cues, such as stress and diet, these epigenetic marks may represent an interface between the genome and environment that could program risk for the development of T2DM and obesity.

From genome-wide studies [10–14], we selected a panel of candidate genes known to be involved in glucose regulation and insulin resistance. Among them, Caveolin-1 (*CAV1*) has been pointed out as one of the genes upregulated in fat tissue of rats in response to a high-fat diet [15] and shown to be involved in insulin resistance [16]. *CAV1* is considered an essential element of adipocyte caveolae and has been shown to be involved in the compartmentalization and integration of several signal transduction pathways in these cavities [17].

We herein set out to investigate how a lifestyle change programme may influence the genetic and epigenetic determinants linked with weight loss after a lifestyle intervention programme and we compared the lifestyle change with an invasive and intensive change, i.e. bariatric surgery. In light of this, the aim of this study was to evaluate the alterations in the expression of a panel of genes, including *CAV1*, implicated in the modulation of weight change and insulin resistance. Additionally, we evaluated for those genes for which we found altered expression with the intervention, if there are associated changes in the DNA methylation.

## 2. Material and methods

### 2.1. Sample collection

Patients with IGR (10 females and 10 males) living in Salford were recruited from November 2014 to November 2018 and underwent anthropometric measurements including waist measurement and waist–hip ratio before the start of the 6-month intervention, and after its completion.

The study recruited individuals with IGR (cases) through Care Call as well as healthy, age, sex and BMI matched individuals (as controls) through Citizen Scientist Website at Salford Royal NHS Foundation Trust. For the present study, we are showing only the results regarding the cases in two different points: before and after an intervention, characterizing as a prospective interventional study. The IGR individuals have been referred to the Salford Care Call intervention by their GP, following administration of a standard Oral Glucose Tolerance Test (OGTT) or with an abnormal fasting glucose (>6 mmol/L) along with raised glycosylated haemoglobin (Hba1 C of >42 and <48 mmol/mol). Care Call is an established telephone-based service in Salford, aimed at improving the glucose control of individuals with IGR by providing guidance on lifestyle, healthy diet and regular exercise. The CareCall and Eatwell guide are provided as a supplementary material (S1).

Inclusion/exclusion criteria: Inclusion Criteria (cases): (i) Patients with IGR (as defined in paragraph 2); (ii) Age 18–80 y. Exclusion criteria (cases): (i)Age <18 y or >80 y; (ii) Unable or unwilling to provide informed consent; (iii) Individuals on long-term (>3 months) steroid therapy; (iv) Pregnancy; (v) Cushing’s syndrome or active acromegaly.

All individuals participated in the Care Call Programme. This is a modular intervention programme which utilizes motivational support techniques, such as lifestyle education, one-to-one and peer discussion and encouragement of progress with goals and signposting/referral to relevant services, also offering tailoring of content to individual needs.

Salford Royal’s Diabetes Care Call is a telephone-based service available to help people with diabetes and has been further developed to also support people diagnosed with IGR presenting a high risk to develop T2DM.

The Care Call programme is structured by offering to the patients eight appointments by telephone with personal and dietary advices; information about diet, lifestyle and activity including tips that can help the patients to reduce the risk to develop T2DM.

Additionally, an ‘Eatwell guide’ is offered to help the patients to understand which types of food they should aim to eat each day and what portion size should be. In addition, the health advisors incentive the patients to be more active, when patients are suitable the advisors can refer them to any Fit City Centre located in Salford and offer a free 8 weeks pass.

We also collected fat tissue and blood at two time points, before and 6 months after the lifestyle intervention. The complete description of samples collecting was described before by our group [7].

This study was submitted to the Ethics committee of Research and Development Department of Salford Royal NHS Foundation Trust and the permission was granted in accordance with the Research Governance Framework (2005), Medicines for Human Uses (Clinical Trials) Regulations (2004) and Salford Royal NHS Foundation Trust local policies (proc number 14/NW/1196).

We also recruited 12 obese patients (n = 5 with T2DM and n = 7 without T2DM) waiting for bariatric surgery at Salford Royal Hospital (Salford, UK). The smoker’s patients were required to stop smoking from at least 2 weeks before to the surgery date. All participants attended The Wellcome Trust Clinical Research Facility (Manchester, UK) where they had taken subcutaneous fat tissue samples at baseline and 6 months after the surgery (n = 10 were submitted to Roux-en-Y gastric bypass and n = 2 to laparoscopic sleeve gastrectomy).

All the following experiments for gene expression and DNA methylation were conducted in a blind way to reduce or eliminate experimental biases. NM and GC participated in the recruitment of IGR participants, sample collection and labelling. SA participated in the recruitment, sample collection and labelling of bariatric surgery patients. HAF and KS conducted all the molecular biology experiments.

### 2.2. Gene expression analysis

All IGR participants and controls had 5 ml of peripheral blood (PAXGene tubes) and both IGR, controls and bariatric surgery patients had a punch of subcutaneous fat tissue collected for subsequent DNA and RNA extraction. Genomic DNA and RNA were extracted using AllPrep DNA/RNA Mini Kit (Qiagen, Valencia, CA). Gene expression analyses were conducted for 18 genes in fat tissue (*PPARG, GIPR, IGF2BP2, FTO, CAV1, IGF1 R, INSR, IGFBP4, WFS1, IGF1, LPL, IGFBP2, IGF2, IGFBP6, LEP, LDLR, IGFR2, HHEX)*, known to be implicated in the modulation of weight change and interventions as diet and exercise, impaired glucose regulation and type 2 diabetes. Total RNA was extracted using the All prep DNA/RNA mini kit (Qiagen, Valencia). The viability and quantity of the RNA were determined by NanoDrop® ND-1000 spectrophotometer (Nanodrop, Wilmington, DE). High-Capacity cDNA Reverse Transcription Kit (Life Technologies, Foster City, CA) was used to synthesize cDNA by using approximately 400 ng of each RNA sample, and 100 ng was then diluted in H_2_O.

Relative gene expression was determined using a LightCycler 480 machine (Roche) running LightCycler 480 SW 1.5.0 SP3 software. The assays used in this study were Roche RealTime ready single assays (*PPARG, GIPR, IGF2BP2, FTO, CAV1, IGF1 R, INSR, IGFBP4, WFS1, IGF1, LPL, IGFBP2, IGF2, IGFBP6, LEP, LDLR, IGFR2, HHEX*) relative to two housekeeping genes (*ACTB* and *RN18S1*). All genes were assayed in triplicate and 50 ng of total cDNA was used per reaction for all. The PCR protocol was as follows: Pre-incubation – 1 cycle at 95ºC for 10 min, Amplification – 50 cycles at 95ºC for 10 s, 60ºC for 30 s and 72ºC for 1 s, cooling – 1 cycle at 40ºC for 30 s. From those 18 genes analysed, we found significant alteration only in *CAV1* in fat tissue, so we also proceed to analyse the expression in buffy coat following the same protocol.

Lymphoprep™ (Axis-Shield PoC AS, Oslo, Norway) was used to obtain the mononuclear cells. The blood samples were diluted 1:1 with physiological saline before being applied to the gradient. The tubes containing Lymphoprep™ and blood diluted in saline were centrifuged at 800× g for 10 min at room temperature (15–25°C) with the brake off. The concentrated leukocyte band (buffy coat) was removed using a Pasteur pipette and used later to extract DNA and RNA.

We quantified the gene expression using the Comparative threshold (Ct) method (ΔΔCt method) [18,19], and the amount of target gene was normalized to *ACTB* as housekeeping gene (as it was stable across all samples) and determined by 2^−ΔΔCt^, as previously described [20,21], with relative expression levels reported as fold change. Ct values higher than the cut-off of 35 were not considered as a reliable expression value, according to the manufacturer’s recommendations, and therefore were excluded from the statistical analysis.

### 2.3. DNA bisulphite conversion and pyrosequencing

The DNA of all IGR patients was bisulphite-treated to convert unmethylated cytosine residues to uracil, we performed this step using the EpiTec Fast DNA Bisulphite Kit (Qiagen) with 99% mean conversion.

**Figure 1.**
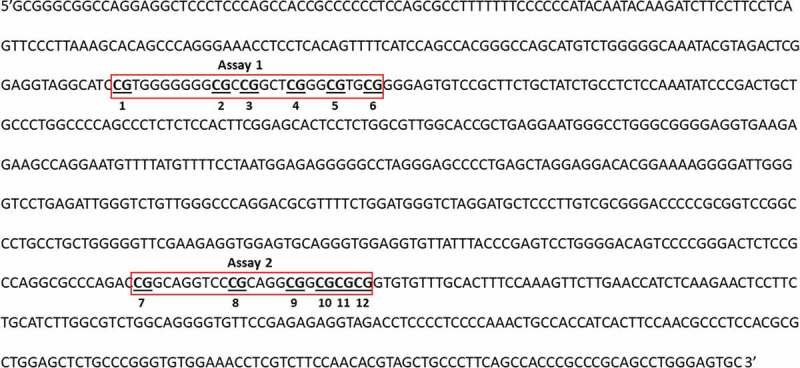
Promoter region of Caveolin-1 (CAV1: Chromosome 7, NC_000007.14 (116524785.116561185)) showing the CpGs chosen to be analysed (numbered 1 to 12 and underlined) and the location of each assay (Assay 1 and 2, red boxes) developed to pyrosequencing those CpG targets. Results were calculated by Paired T-test and are shown as average ± SE. * p ≤ 0.025

As we only found differences related to the lifestyle intervention in gene expression for CAV1, DNA sequences for CAV1 gene were identified in the 5ʹ region that contains important transcription factor (TF)-binding site sequences (using ALLGEN-PROMO website: http://alggen.lsi.upc.es/cgi-bin/promo_v3/promo/promoinit.cgi?dirDB=TF_8.3), and a pyrosequencing method was developed for determination of methylation at 12 CpG sites within those sequences following bisulphite reaction. The CpGs chosen to analyse are identified in Figure 1.

A measure of global methylation *LINE-1* was determined using the set of primers PyroMark Q24 CpG LINE-1 Catalogue no. 970012 (Qiagen, UK).

PCR reactions were carried out with 20 ng bisulphite-converted DNA using the PyroMark PCR kit in a final volume of 12 µl containing 6 µl 1x PyroMark PCR Master Mix, 1.25 µl 1x CoralLoad Concentrate, 0.5 µl of each primer in a final concentration of 0.05 µM, 4 µl RNase-free water. Amplification conditions were done as follows: 95°C for 15 min, 45 cycles of 94°C for 30 s, 56°C for 30 s and 72°C for 30 s, finally, 72°C for 10 min. The methylation levels in the promoter sequence of CAV1 were determined with a PyroMark Q48 pyrosequencer (Qiagen UK) using 10 µl of PCR product and a sequencing primer. Pyrosequence setup and data reading were conducted by PyroMark Q48 2.4.2 software. We carried out our PCR and pyrosequencing experiments in duplicate; any inconsistencies between samples were resolved following further repetition. The set of primers designed to analyse CAV1 promoter gene is described in Table 1.

**Table 1.** Set of primers used for PCR (forward and biotinylated reverse) reactions and pyrosequencing (sequencing) of CAV1 promoter

Primers (5ʹ–3ʹ)	Assay 1	Assay 2
Forward	GGTTAGTATGTTTGGGGGTAAAT	TGAGATGGTTTAAGAATTTTGGAAAGTGTA
Reverse [BIO]	ACATAAAACATTCCTAACTTCTCTT	CCTAAAAAACCCCTAAACTAAAAAAACAC
Sequencing	GTAGATTAGGAGGTAGGT	TTAAGAATTTTGGAAAGTGTAA

### 2.4. Statistical analysis

We have done comparisons between patients pre- (baseline) and post-intervention using paired T-test. Statistical analysis was performed using the ‘Statistical Package for Social Sciences’ (SPSS) version 22.0 (IBM Corp: Armonk, NY: USA). We used p-value adjusted for multiple comparisons (p ≤ 0.025). Age and sex were used as covariates.

We also investigated possible correlations between gene expression/DNA methylation and body measurements as BMI, hip/waist ratio and weight, and between gene expression and DNA methylation using partial correlation (Pearson’s), p ≤ 0.05 were considered significant and r coefficient interpreted according to Mukaka (2012) and Schober et al. (2018) [22,23].

## 3. Results

The anthropometric measurements and demographic data taken of the IGR patients before and after the intervention were published in our previous study [7]. This clinical trial ended in 2018 due to adjustments in the Care Call programme. Data regarding patients submitted to bariatric surgery are shown in Table 2.

**Table 2.** Anthropometric measurements and demographic data of patients submitted to bariatric surgery (n = 12)

		Before	After (*p < 0.025)
*Age (years ± SD)*	53.5 ± 6.7		
Sex (% male)	0.08		
BMI (Average ± SEM)		49.2 ± 2.3	36.3* ± 2.1 (p < 0.001)
Waist (cm)		133.1 ± 1.9	108.9* ± 3.8 (p < 0.001)
T2DM history (%)	41		
Weight (kg, average ± SEM)		130.5 ± 5.6	98.5*±5.8 (p < 0.0001)

### 3.1. Gene expression

Amongst all the genes analysed, only *CAV1* showed differences related to the lifestyle intervention (Table 3).

**Table 3.** Panel of genes analysed by RT-qPCR in fat tissue of impaired glucose regulation (IGR) patients (n = 20) before and 6 months after the lifestyle intervention. The results are shown as average ± SE. * p ≤ 0.025

	Fold change mean ± SE			p value
Gene	*(Before)*	*(After)*	T value (paired T-test)	(*p ≤ 0.025)
PPARG	2.76 ± 0.9	1.62 ± 0.3	1.132	0.273
GIPR	2.96 ± 1.2	3.10 ± 0.6	−0.124	0.903
IGF2BP2	3.30 ± 1.3	2.30 ± 0.5	0.683	0.504
FTO	3.20 ± 0.7	1.81 ± 0.3	1.969	0.065
CAV1	1.85 ± 0.2	1.04 ± 0.1	2.263	0.025*
IGF1 R	1.70 ± 0.5	1.65 ± 0.4	0.066	0.948
INSR	1.70 ± 0.3	1.78 ± 0.5	−0.246	0.809
IGFBP4	3.30 ± 1.0	2.89 ± 0.6	0.431	0.672
WFS	2.91 ± 0.8	2.21 ± 0.5	0.820	0.424
IGF-I	2.01 ± 0.5	1.95 ± 0.4	0.089	0.930
LPL	9.90 ± 3.2	7.50 ± 2.0	0.817	0.426
IGFBP-2	1.65 ± 0.4	1.22 ± 0.1	1.161	0.262
IGF-II	3.71 ± 1.4	3.18 ± 0.8	0.451	0.658
IGFBP-6	2.60 ± 1.0	2.26 ± 0.4	0.338	0.739
LEP	15.4 ± 5.2	14.6 ± 4.6	0.124	0.903
LDLR	2.43 ± 1.0	1.04 ± 0.2	1.458	0.163
IGFR-2	2.30 ± 0.6	1.97 ± 0.4	0.759	0.458
HHEX	2.34 ± 0.5	2.22 ± 0.6	0.267	0.793

**Figure 2.**
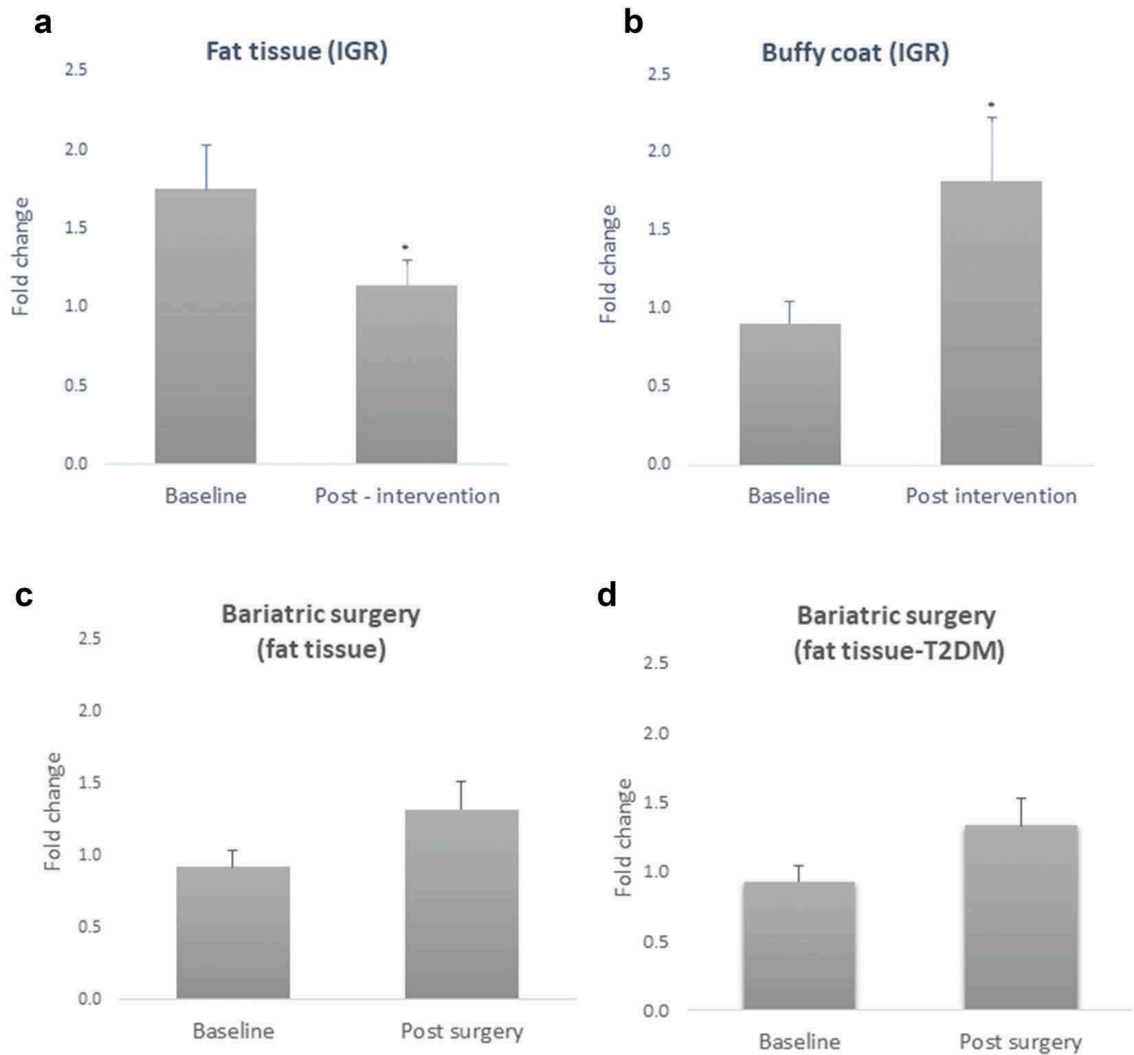
Gene expression of Caveolin-1 (*CAV1*) in fat tissue (a); buffy coat (b) from patients with impaired glucose regulation (IGR) at baseline and after a lifestyle change (6 months post-intervention); (c) fat tissue from patients submitted to bariatric surgery at baseline and 6 months post-surgery; (d) fat tissue from patients with T2DM history submitted to bariatric surgery at baseline and 6 months post-surgery. Results were calculated by Paired T-test and Bonferroni correction, and are shown as average ± SE. * p ≤ 0.025

**Figure 3.**
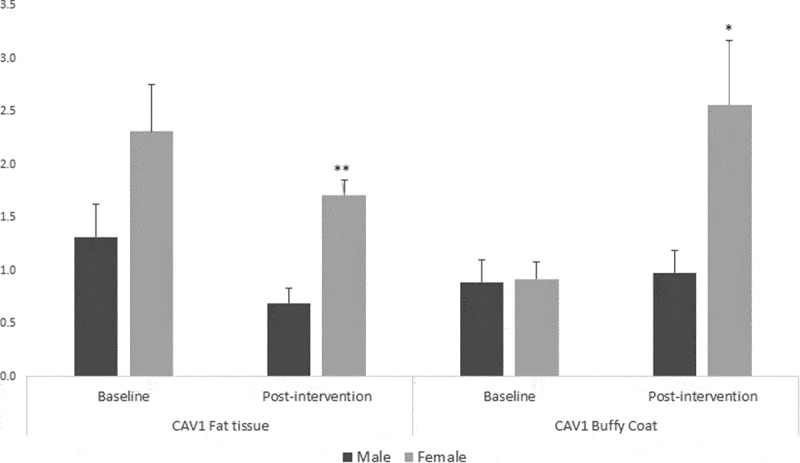
Sex differences in Caveolin-1 (*CAV1*) gene expression in fat tissue and buffy coat of patients with impaired glucose regulation (IGR) at baseline and after a lifestyle change (6 months post-intervention). Results were calculated by Paired T-test and are shown as average ± SE. * p ≤ 0.025, ** p ≤ 0.01

The results related to gene expression in IGR individuals of *CAV1* in both fat tissue and buffy coat blood cells are shown in Figure 3. We found that the lifestyle intervention (6 months) inhibited the expression of *CAV1* in fat tissue (t = 2.26, p = 0.025, n = 20) (Figure 2(a)) while its expression was increased in buffy coat blood cells (t = −2.76, p = 0.015, n = 20) (Figure 2(b)).

Regarding those patients submitted to bariatric surgery, we did not find significant differences in *CAV1* expression before and after surgery (t = – 0.744; p = 0.473, n = 12) (Figure2(c)). Considering patients with T2DM history, we found a discreet increase of *CAV1* expression after the surgery, however not achieving statistical significance (t = – 2.28, p = 0.084, n = 5) (Figure 2(d)).

Additionally, the general linear model analysis showed gender differences in gene expression for *CAV1* in both fat tissue and buffy coat after the lifestyle intervention (F = 18.6, p = 0.002 and F = 9.96, p = 0.012, respectively) with a similar pattern seen at baseline; women showed higher levels of *CAV1* than men in both cases (t = 5.04, p = 0.0001 in fat tissue and t = 2.46, p = 0.034 in buffy coat, n = 10) (Figure 3).

### 3.2. DNA methylation

As we found gene expression changes in *CAV1* induced by the lifestyle intervention in IGR individuals, we investigated the DNA methylation profile in these samples. We analysed 12 CpG sites within the promoter region of *CAV1* gene (Chromosome 7, NC_000007.14 (116524785.116561185)) and we found tissue-specific changes in DNA methylation.

**Figure 4.**
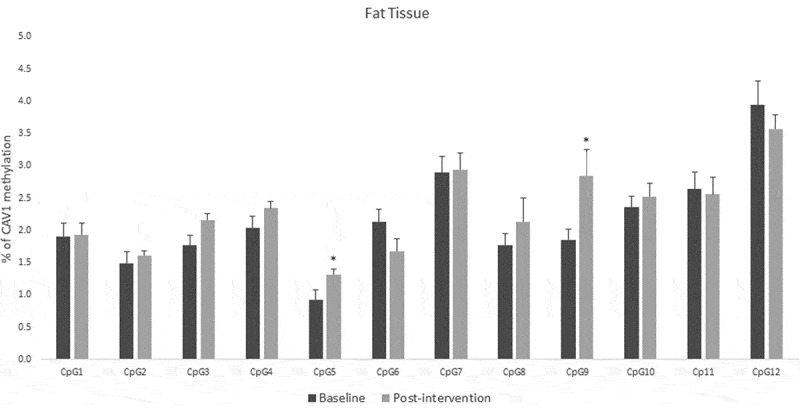
DNA methylation levels at 12 CpG sites within the promoter region of Caveolin-1 (*CAV1*) in fat tissue of patients with impaired glucose regulation (IGR) at the baseline and after 6 months of lifestyle change. Results were calculated by Paired T-test and are shown as average ± SE. * p ≤ 0.025

**Figure 5.**
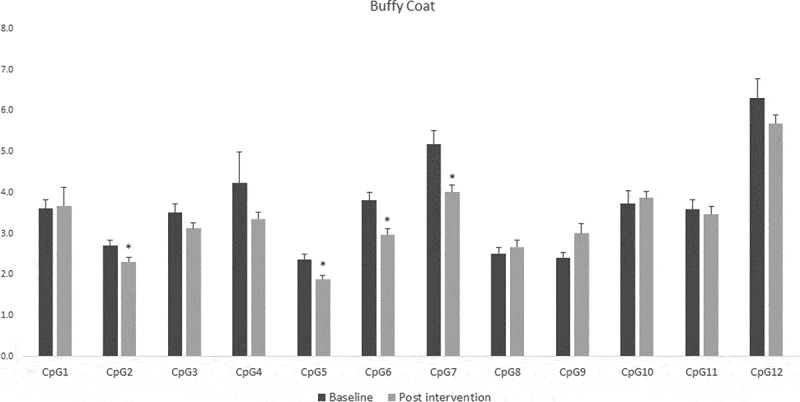
DNA methylation levels at 12 CpG sites within the promoter region of Caveolin-1 (*CAV1*) in buffy coat of patients with impaired glucose regulation (IGR) at the baseline and after 6 months of lifestyle change. Results were calculated by Paired T-test and are shown as average ± SE. * p ≤ 0.025

**Figure 6.**
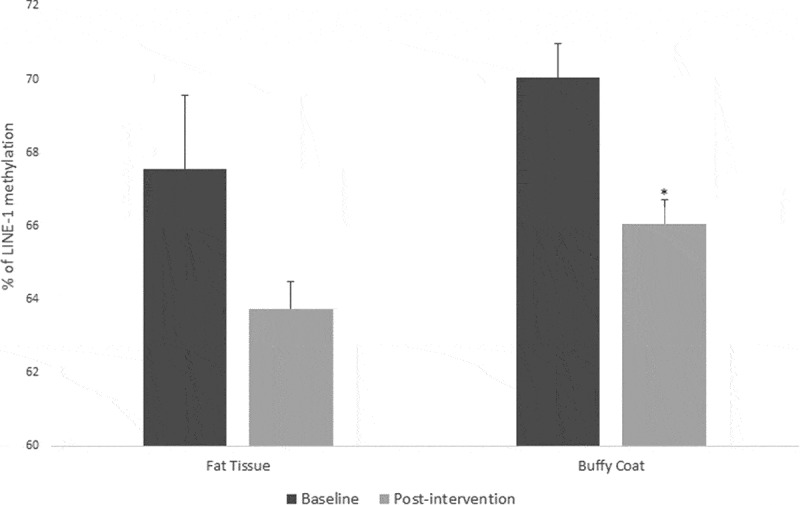
LINE-1 methylation in fat tissue and buffy coat of patients with impaired glucose regulation (IGR) at the baseline and after 6 months of lifestyle change. Results were calculated by Paired T-test and are shown as average ± SE. * p ≤ 0.025

For fat tissue we found increased methylation in 2 CpG sites related to lifestyle intervention (CpG5: t = – 2.39, p = 0.025 and CpG9: t = – 2.66, p = 0.017), these changes are shown in Figure 4. While for buffy coat blood cells the overall effect was the opposite – the majority of the CpGs showed decreased levels of methylation after the intervention (CpG2: t = 2.58, p = 0.021, CpG5: t = 3.72, p = 0.002, CpG6: t = 5.53, p = 0.0001, CpG7: t = 3.38, p = 0.004) (Figure 5). *LINE-1* methylation analysis showed differences only in buffy coat blood cells after the intervention (t = 3.46, p = 0.003) (Figure 6).

### 3.3. Correlations

In order to account for the global *LINE-1* methylation observed in buffy coat, all the correlations were analysed by partial correlation and controlling for *LINE-1*.

**Figure 7.**
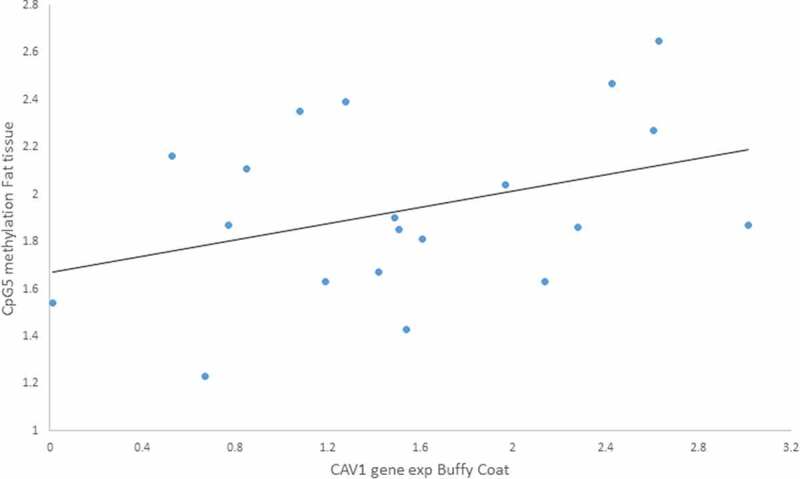
Positive Pearson correlation between CpG5 of fat tissue and Caveolin-1 (*CAV1*) expression in buffy coat of patients with impaired glucose regulation (IGR) after the lifestyle intervention. r = 0.617, p = 0.043

**Figure 8.**
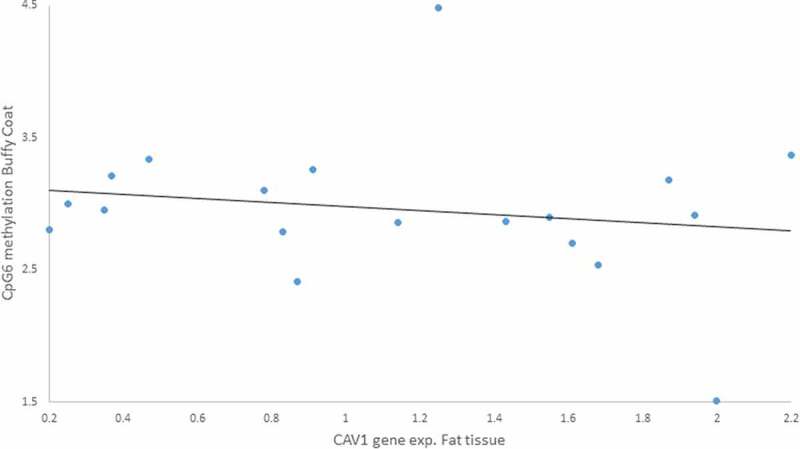
Negative Pearson correlation between CpG6 of buffy coat and Caveolin-1 (*CAV1*) expression in fat tissue of patients with impaired glucose regulation (IGR) after the lifestyle intervention. r = – 0.606, p = 0.028

We tested Pearson’s correlations between those CpGs we found altered due to the lifestyle intervention and the anthropometric measurements for both tissues. There were associations between gene expression and methylation: i) a positive moderated correlation between *CAV1* gene expression in buffy coat and CpG5 methylation in fat tissue (r = 0.617, p = 0.043) (Figure 7), ii) a negative moderated correlation between *CAV1* gene expression in fat tissue and CpG6 methylation in buffy coat (r = – 0.606, p = 0.028) (Figure 8).

**Figure 9.**
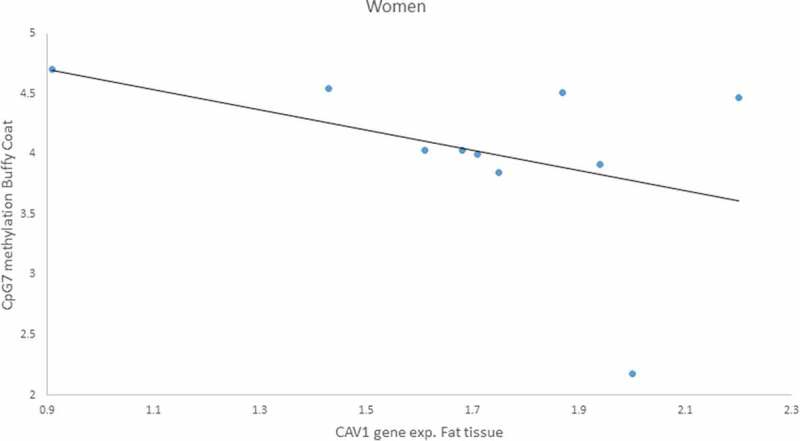
Negative Pearson correlation between CpG7 of buffy coat and Caveolin-1 (*CAV1*) expression in fat tissue of women patients with impaired glucose regulation (IGR) after the lifestyle intervention. r = – 0.959, p = 0.041

Furthermore, because we found a sex difference in gene expression for both buffy coat and fat tissue, we analysed the correlation by gender and found a negative correlation only in women of *CAV1* gene expression in fat with CpG7 methylation in buffy coat (r = – 0.959, p = 0.041) (Figure 9).

**Figure 10.**
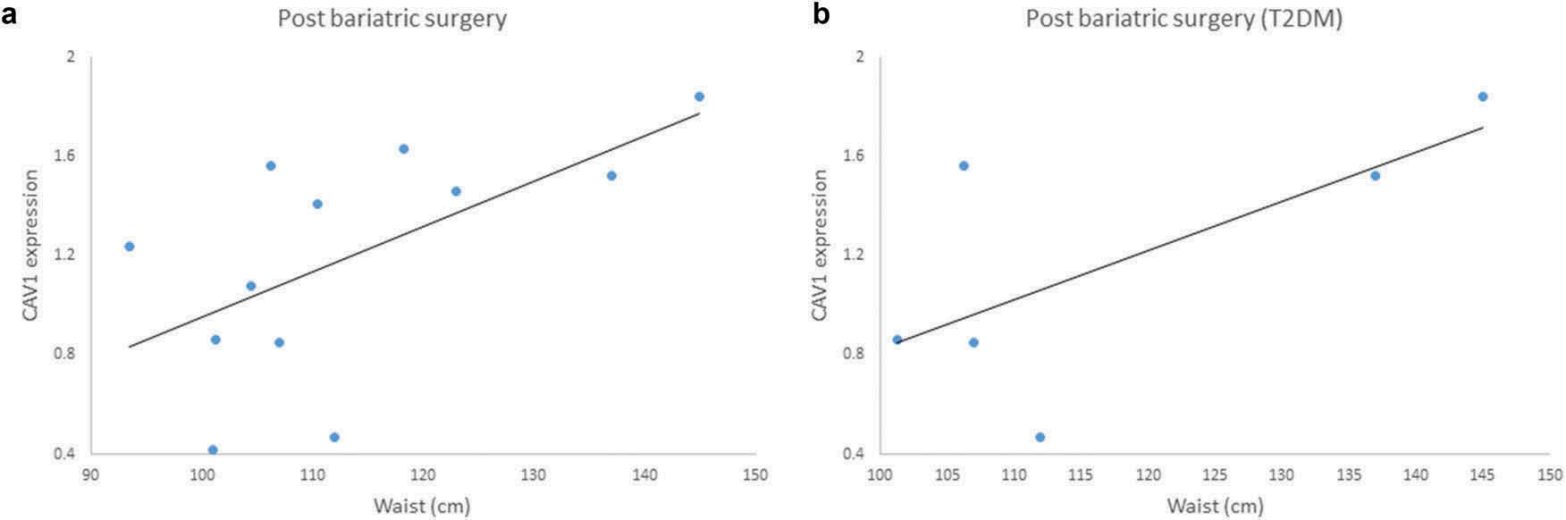
(a) Positive correlation between Caveolin-1 (*CAV1*) gene expression and waist measurements in patients submitted to bariatric surgery (6 months post-surgery) r = 0.600; p = 0.039. (b) Positive correlation between *CAV1* gene expression and waist measurements in patients with T2DM history submitted to bariatric surgery (6 months post-surgery), r = 0.931; p = 0.021

We also found a positive moderated correlation between *CAV1* expression and waist measurements in patients after the bariatric surgery (r = 0.600, p = 0.039) (Figure 10(a)) and this correlation is even stronger when considering only patients with T2DM history (r = 0.931, p = 0.021) (Figure 10(b)).

## 4. Discussion

In this study, we evaluated how a lifestyle intervention followed by weight loss could influence the gene expression and DNA methylation profile in fat tissue and buffy coat from patients with IGR and we compared these results with patients submitted to bariatric surgery.

Our main findings are that the lifestyle intervention resulted in changes in gene expression and DNA methylation of *CAV1* in a tissue-specific way. Additionally, we found correlations between gene expression and DNA methylation, suggesting crosstalk between tissues. Furthermore, we found a correlation between *CAV1* expression and waist measurements after bariatric surgery, suggesting that *CAV1* expression follows the same direction of abdominal fat deposition. In other words, when the waist circumference increases the CAV1 expression in fat tissue increases, as well as when the waist reduces, the CAV1 expression also reduces.

Caveolin-1 (*CAV1*), the main structural and functional protein of caveolae, is a key mediator of the insulin transduction pathway directly interacting with the beta-subunit of insulin receptor (IR) [24]. *CAV1* plays an essential role for a proper insulin response in mature adipocytes, since its depletion causes insulin resistance and glucose transporter-4 (Glut-4) degradation [25,26]. The opposite direction of changes in *CAV1* expression in fat tissue and blood after the intervention is reflecting the weight reduction, suggesting a compensatory mechanism resulting in less signal in fat tissue followed by increased expression in blood. The maintenance of *CAV1* levels is important for a proper insulin response and lipid storage as demonstrated before that *CAV1* null mice presented altered lipid deposition and improper storage of lipids outside of the adipose tissue [27].

*CAV1* has also been associated with immune response and inflammation; it has been shown that *CAV1* expression in fat tissue of T2DM patients is linked with low-grade inflammation [28,29] stimulating the production of major pro-inflammatory cytokines, such as TNF-α, IL-6, IL-1. On the other hand, reduced expression of Caveolin-1 in monocytes was inversely correlated with Toll-like receptor (TLR) 4 levels in T2DM and neuropathy patients suggesting that low *CAV1* expression in white cells could aggravate the TLR4-mediated inflammatory cascade [30]. Evidence has suggested that *CAV1* plays an important part in suppressing inflammation. It was has been shown that upregulation of Caveolin-1 in murine macrophages dramatically inhibits TNF-α and IL-6 production [31].

The interaction between the methylation and gene expression across tissues also suggests that the methylation changes may underlie the expression of *CAV1* due to external factors, in this case, the lifestyle intervention. It was previously shown that DNA methylation of the promoter can control the *CAV1* expression during adipocyte differentiation and this process was accompanied by activation of IR and glucose uptake [32]. The activation of *CAV1* in peripheral blood may be crucial for the maintenance of the equilibrium in the insulin-response efficiency and controlling immune response.

We also found a gender association with *CAV1* expression; women expressed more *CAV1* than men post-intervention. Even though we did not find studies showing higher levels of *CAV1* in adipose tissue in women, previous evidence demonstrated a role for *CAV1* in regulating sex hormone signalling [33]; furthermore, it was demonstrated that *CAV1* expression is stimulated by 17-b-oestradiol (E2) and reduced by dihydrotestosterone (DHT) in both in vitro and in vivo studies [34], showing these hormones have opposite effects on body weight parameters.

The CpGs chosen in this study are important binding sites for transcription factors (TF) that regulate gene expression. It is important to highlight that at CpGs 5 and 6 (those we found correlated to gene expression) there is a binding site for aryl hydrocarbon receptor (AhR). AhR is a ligand-activated TF which has been associated with an organism’s response to environmental contaminants and may perturb endocrine function [35]; for example, AhR-dependent chemicals (as dioxin) downregulate the expression of PPARγ in 3T3 cells during adipogenesis, resulting in the inhibition of adipocyte differentiation [36]. Thus, the differential methylation pattern seen in fat and blood may participate in the regulation of adipocyte differentiation.

At CpG7 there is a TF-binding site for CAAT/enhancer-binding protein β (C/EBPβ) which is involved in the coordination of gene expression in adipose [37] and crucial to adipogenesis process [38]; additionally, its function is enhanced by DNA methylation [39]. We found that this binding site is hypomethylated in buffy coat and correlated with increased *CAV1* gene expression in fat tissue in women, revealing the inhibition of C/EBPβ activity by decreased methylation.

Reduced levels of global DNA methylation (*LINE-1*) have been associated with genomic instability and susceptibility to chronic diseases [40] and frequently used as a control of epigenetic changes. The lifestyle intervention caused a reduction in *LINE-1* methylation in buffy coat blood cells accompanied by similar changes in fat tissue however not achieving significance. *LINE-1* methylation has previously been associated with dietary intake. Individuals presenting with higher *LINE-1* methylation had higher daily intakes of calories [41]. Lifestyle and dietary intake changes may have a role in the regulation of *LINE-1* methylation in blood cells. It is important to highlight that our *CAV1* methylation findings are independent of *LINE-1* changes, showing that the lifestyle intervention modulated a specific effect of this gene.

Interestingly *CAV1* expression changes were more robust after the lifestyle intervention than after bariatric surgery in our study. This evidence suggests that *CAV1* gene expression can be better modulated by an improvement in lifestyle, such as healthier food consumption and exercises than due to a more invasive procedure.

Our study has some limitations due to the number of participants, even though we have strength in that participants consented to donate blood and fat tissue pre- and post-intervention, which permitted us to identify tissue-specific responses after diet and exercise in a timescale.

This is the first evidence showing tissue-specific gene expression and DNA methylation changes in the *CAV1* promoter gene in patients with IGR, accompanied by gender differences in *CAV1* expression. Our findings suggest a role for *CAV1* in modulating adipocyte function as a consequence of diet and exercise. These results may underlie the mechanisms related to the T2DM prevention and provide insights into new therapeutic targets for diabetes prevention.

## Supplementary Material

Supplemental Material
