# Supplementary material for: Lifestyle intervention in individuals with impaired glucose regulation affects Caveolin-1 expression and DNA methylation
Source: Adipocyte. 2020 Mar 3;9(1):96–107. doi: 10.1080/21623945.2020.1732513 (PMC7153542; doi:10.1080/21623945.2020.1732513)
Supplement: Supplemental Material [file KADI_A_1732513_SM1093.pdf]

HEALTHIER YOU
NHS DIABETES PREVENTION PROGRAMME

Salford Royal NHS
NHS Foundation Trust

University Teaching Trust

safe • clean • personal

Preventing type 2 diabetes the NDH Care Call Service

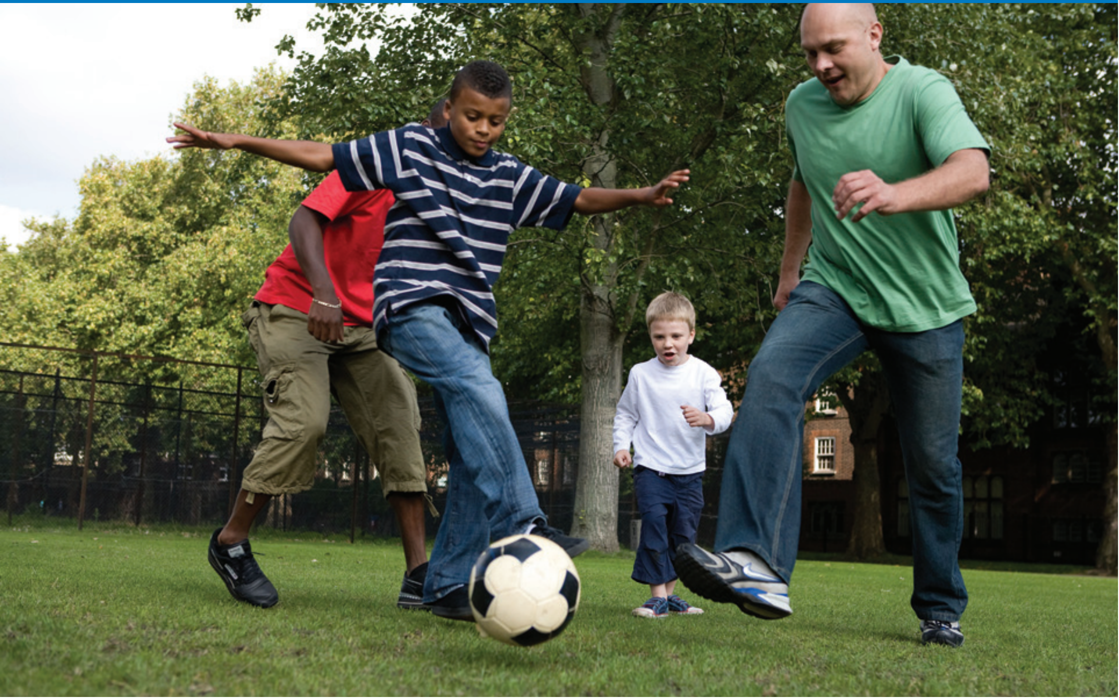

Diabetes Care Call Team
0161 206 5668

Health & care information you can trust
The Information Standard Certified Member

© G17062301W. Design Services, Salford Royal NHS Foundation Trust, All Rights Reserved 2017.
Document for issue as handout. Unique Identifier: MED35(17). Review date: July 2019

Being at risk of type 2 diabetes

In Salford, approximately 12,000 people have diabetes and of those around 90% (10,800) have type 2 diabetes. Diabetes is a serious condition and if not properly controlled can lead to serious complications which have major implications for health and quality of life.

Some people do not know they have diabetes and could have had it for months or years without knowing. This is because the condition often develops gradually without any warning signs or symptoms. Often it is only when the GP takes a blood sample they find out they have diabetes.

At least 19,600 people in Salford have Non-Diabetic Hyperglycaemia (NDH) and 50% of these will progress to type 2 diabetes over the next 5-10 years.

What is NDH?

You recently had a blood test called **HbA1c** and the results of this blood test have shown that your glucose levels are higher than normal but not quite high enough to be diagnosed with type 2 diabetes. This means you have a condition called NDH (Non-Diabetic Hyperglycaemia).

Most people produce the hormone called insulin in our pancreas.

Insulin lowers the level of glucose in the blood by taking it from the blood stream and moving it to our body cells where we need it for energy.

When you have NDH it means your body is not producing enough insulin, or that your body is not using the insulin very well (this is called insulin resistance). If the insulin is not taking glucose from the bloodstream very well the amount of glucose in your blood starts to rise above normal level. This is what has been picked up at your **HbA1c** blood test.

Have I got diabetes?

No, although you have been diagnosed with NDH this does **NOT** mean you have type 2 diabetes, it means you have an **'increased risk'**.

However, although you may not experience any symptoms of NDH it still means you have raised sugar levels which is a serious condition as it also increases your risk of **heart disease, stroke** and other **cardiovascular diseases**.

The good news is that now we know you have NDH we know you can reduce your risk of developing type 2 diabetes and other cardiovascular diseases by making simple lifestyle changes.

Why am I at risk of developing type 2 diabetes?

There are many risks that can lead to type 2 diabetes:

- If you are white and over the age of 40 years
- If you are black or South Asian and over the age of 25 years

and if you have one (or more) of the following:

- If you have previously been diagnosed with NDH
- If you are overweight or obese (BMI >25 Caucasian; BMI >23 South Asian).
- If your waist is over:
  - 31.5 inches (80cm) or over for all women
  - 35 inches (89cm) for south Asian men
  - 37 inches (94cm) or over for white or black men

continued next page

© G17062301W. Design Services, Salford Royal NHS Foundation Trust, All Rights Reserved 2017. Document for issue as handout. Unique Identifier: MED35(17). Review date: July 2019.

- If you have high blood pressure, or you have had a heart attack or stroke
- If you have a close family member who has type 2 diabetes (such as parent, brother, sister)
- If you have had gestational (pregnancy related) diabetes
- If you are female with polycystic ovary syndrome and are overweight
- If you have severe mental health issues.

Some risk factors we cannot change, for example our family history, our ethnicity or our increasing age.

However, we can do something about the biggest risk factors for developing type 2 diabetes which are, being **overweight** and being **inactive**.

If you have access to a computer why not go to the Diabetes UK website and calculate your risk of developing type 2 diabetes using their risk assessment form.

https://riskscore.diabetes.org.uk

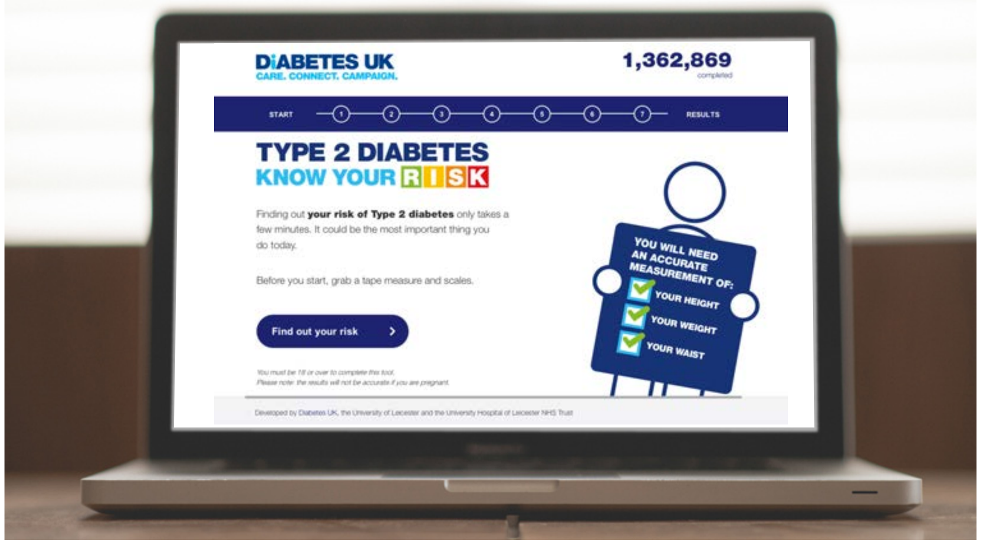

What does my 'raised' HbA1c blood result mean?

The HbA1c blood sample is usually taken from a vein in your arm and you don't have to fast for this sample to be taken.

The result of the HbA1c test tells us what your average blood glucose level has been in the previous 2-3 months.

When you have a raised HbA1c result between 42-47mmol/l this means you have NDH but the good news is we can help you get back down below 42mmol/l (low risk).

Have a look at the chart below and see where your blood test is.

Low risk	NDH	Type 2 diabetes
Less than 42mmol/l	42-47mmol/l	Above 48mmol/l

My results	42	43	44	45	46	47	*Please circle*

© G17062301W. Design Services, Salford Royal NHS Foundation Trust, All Rights Reserved 2017. Document for issue as handout. Unique Identifier: MED35(17). Review date: July 2019.

What can I do?

NDH is a reversible condition! You **CAN** reduce your risk of developing type 2 diabetes.

Research has demonstrated that within the next 10 years up to half the people ‘at risk’ of developing type 2 diabetes will go on to develop it if they don’t make some lifestyle changes.

We know these four key lifestyle changes can reduce your risk of developing type 2 diabetes:

- Keep a healthy weight
- Eat less fat, especially saturated fat
- Eat more fibre
- Be more active

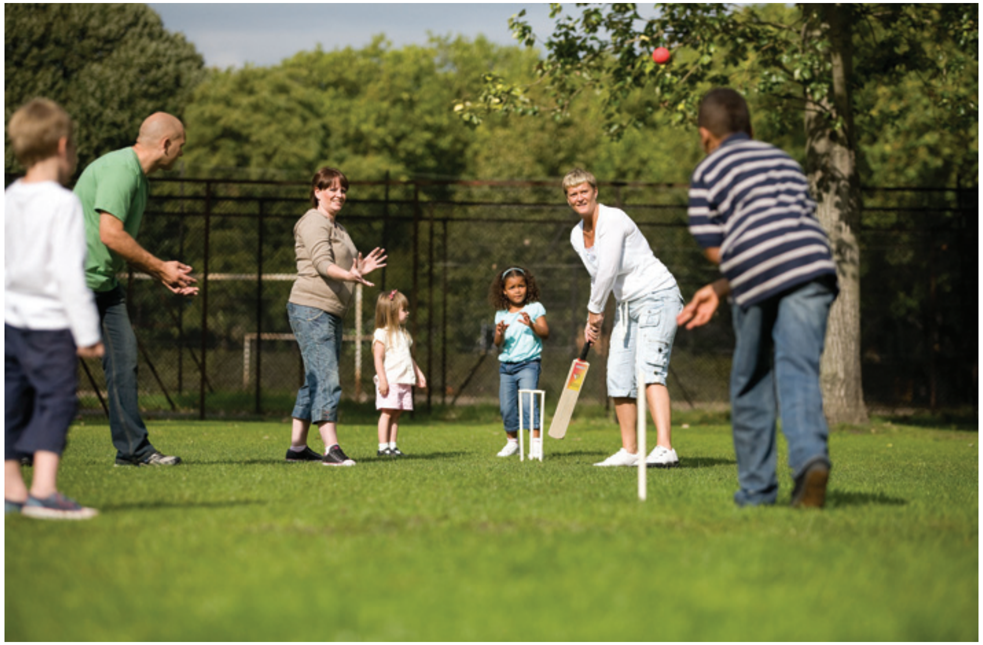

Salford Royal NHS Foundation Trust has a service called Care Call that can help and guide you to make these changes.

What is the Care Call Service and how can it help me?

The Care Call service is a telephone service that offers support to help you make lifestyle changes over a period of nine months.

The Care Call service is accessible and flexible:

- Appointments by telephone to suit you, early morning, throughout the day to 7pm on allocated days.

It is a structured pathway that provides you with:

- Eight monthly appointments by telephone
- Personal advice
- Dietary advice and education
- Relevant literature and resources
- Access to other services in Salford.

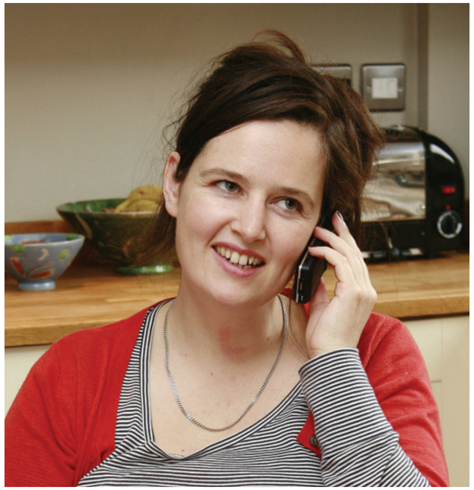

You will be allocated your own Health Advisor who will stay with you throughout all of your calls and will offer individualised support to help you:

- Understand your condition
- Explore your daily lifestyle
- Make small achievable goals.

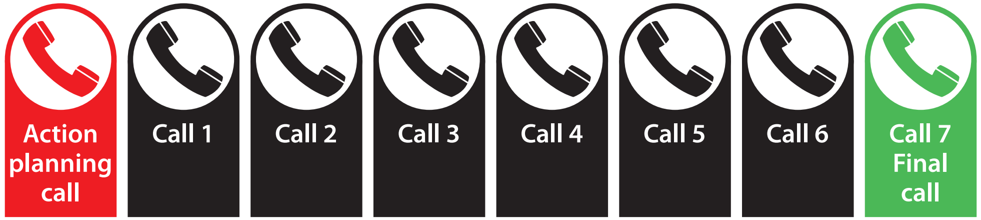

© G17062301W. Design Services, Salford Royal NHS Foundation Trust, All Rights Reserved 2017. Document for issue as handout. Unique Identifier: MED35(17). Review date: July 2019.

What happens during a telephone appointment?

We start with making a telephone appointment for the Action Planning call. This is the longest telephone appointment and will last about 30 minutes. During this telephone appointment we ask you to tell us about your daily routine - this is so we can offer you individualised advice for your lifestyle as no two people are the same!

You will then receive a telephone appointment once a month for six months. Each call will last around 10-15 minutes. During these calls we will give you up to date evidence based information about diet, lifestyle and activity. We offer tips and advice that can help reduce your risk of developing type 2 diabetes.

We give you a final telephone appointment at nine months to ensure you have understood the messages and **give you advice on what to do next.**

What diet and activity advice do you give?

There is no food we will tell you that you **CANNOT** have but we will tell you the best way or time to eat it to help reduce your raised glucose levels!

We discuss the different types of food our body needs, why our body needs food and what it does, portion sizes and when the best time is to eat certain foods.

The advice we give will be tailored to you as an individual and will help reduce your glucose levels back to a normal level.

The Eatwell guide helps us to identify which different types of food we should aim to eat each day and what our portion sizes should be.

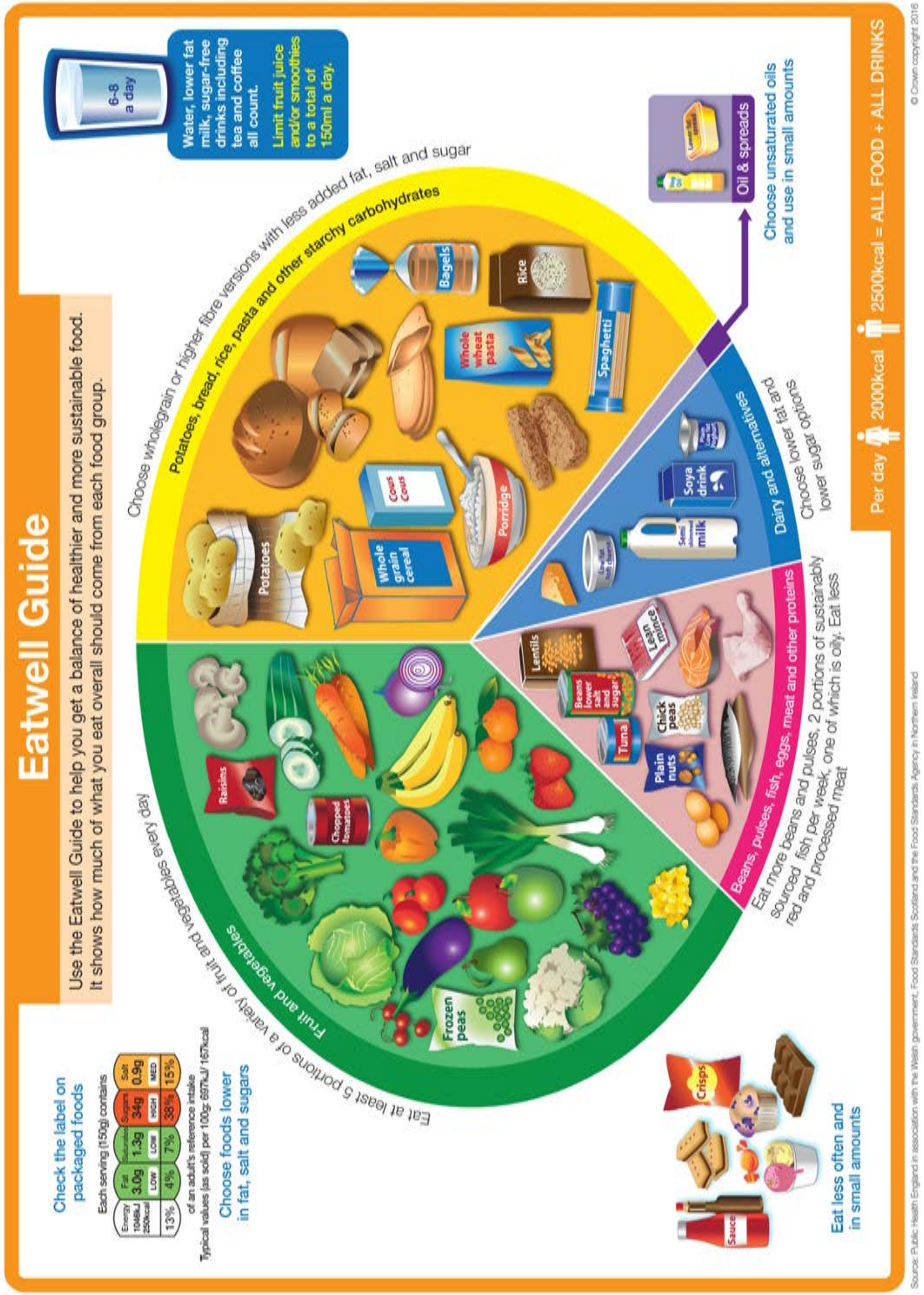

© G17062301W. Design Services, Salford Royal NHS Foundation Trust, All Rights Reserved 2017. Document for issue as handout. Unique Identifier: MED35(17). Review date: July 2019.

We also give you advice on shopping so you can buy your everyday items (sometimes cheaper) that can help reduce your glucose levels and make healthier choices when buying pre-packaged food.

g	LOW	MEDIUM	HIGH
Fat	3g or less	>3g - ≤17.5g	More than 17.5g or >21g/portion
Saturated fat	1.5g or less	>1.5g - ≤5g	More than 5g or >6g/portion
Sugars	5g or less	>5g - ≤22.5g	More than 22.5g or >27g/portion
Salt	0.3g or less	>0.3g - ≤1.5g	More than 1.5g or >1.8g/portion

We can help most people become a little more active, even if you are sat in your armchair watching TV.

We can refer you to any Salford Fit City Centre where you will receive a one off personal session with a trained health coach.

The Health Coach will give you individualised advice and if you are suitable will offer a free 8 week pass to use any Salford Fit City Centre at your leisure.

This means you can use the swimming pool, the gym or even attend any classes that are being held.

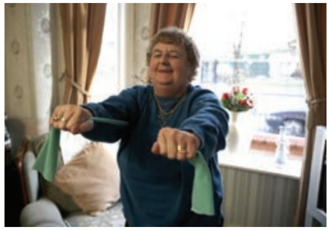

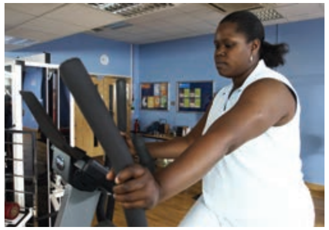

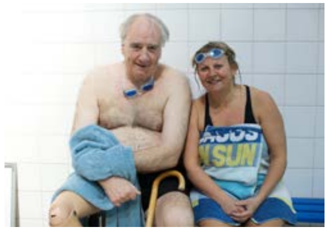

How do I know Care Call can help me?

Recent patient feedback from our service:

“It was much easier to have the calls than to have to go into a clinic, travelling all the way to the doctors surgery or the hospital would have been difficult. I think the Care Call takes the stress out of having to go to those places.”

“The Care Call staff were more caring and understanding and I got better advice from the call staff than I would get from my own doctor”

“We got to know each other a bit and we got a good understanding of each other. You were looking forwards to the phone call”

“She was phoning me every month, we made a time for the call together. It helped to keep me on track each time she phoned, you’re tempted to stray a bit, especially when you’ve got the grandchildren coming”

“It was good that the advice was given over time because it gave me a chance to take in what she was saying”.

“It was easy for me to get the advice on the phone because she fitted in with me rather than me having to fit in with her, we would agree a time and date for the next call that worked for us both”.

“My whole way of eating has changed because of the advice of the Care Call staff. I found it easy because everything they told me to do was doable - I wasn’t dieting I could still eat what I wanted”.

“She was very approachable and didn’t ram things down your throat.

“Some people are a bit ‘“do this, don’t do that” but she wasn’t like that at all, she wanted to listen to what you had to say”

“It definitely made me more aware of what I’m eating, I’ve made a conscious effort to each more vegetables, I’ve always had some but I eat more now. And fruit”

“It was easy to make the changes because they were small changes. I was determined I wasn’t going to become diabetic”

© G17062301W. Design Services, Salford Royal NHS Foundation Trust, All Rights Reserved 2017. Document for issue as handout. Unique Identifier: MED35(17). Review date: July 2019.

Notes

Notes

© G17062301W. Design Services, Salford Royal NHS Foundation Trust,
All Rights Reserved 2017. Document for issue as handout.
Unique Identifier: MED35(17). Review date: July 2019.

Notes

Notes

Contact details

Main Care Call Office

0161 206 5668

healthcoaching@nhs.net

NDH Care Call,
Salford Royal NHS Foundation Trust,
Summerfield House,
Stott Lane,
Salford, M6 8HD

© G17062301W. Design Services, Salford Royal NHS Foundation Trust,
All Rights Reserved 2017. Document for issue as handout.
Unique Identifier: MED35(17). Review date: July 2019.

© G17062301W. Design Services
Salford Royal NHS Foundation Trust
All Rights Reserved 2017
This document **MUST NOT** be photocopied

Information Leaflet Control Policy:

Unique Identifier: MED35(17)
Review Date: July 2019

Salford Royal NHS
NHS Foundation Trust

University Teaching Trust

safe • clean • personal

For further information on this leaflet, it's references and sources used, please contact **0161 206 5668.**

Copies of this information are available in other languages and formats upon request.

In accordance with the Equality Act we will make 'reasonable adjustments' to enable individuals with disabilities, to access this treatment / service.

If you need this interpreting please telephone

Polish
Jeżeli potrzebne jest Państwu to tłumaczenie, proszę zadzwonić pod numer.

Urdu
اگرآپ کواس ترجمانی کی ضرورت ہے تو مہربانی کرکے فون کریں۔

Arabic
اذا كنتم بحاجة الى تفسير او ترجمة هذا الرجاء الاتصال

Chinese
如果需要翻译，请拨打电话

Farsi
اگر به ترجمه این نیاز دارید ، لطفا تلفن کنید

0161 206 0224

Email: InterpretationandTrans@srft.nhs.uk

Salford Royal operates a smoke-free policy.

For advice on stopping smoking contact the Hospital Specialist Stop Smoking Service on 0161 206 1779

Salford Royal NHS Foundation Trust
Stott Lane, Salford,
Manchester,
M6 8HD
Telephone 0161 789 7373
www.srft.nhs.uk

If you would like to become a Foundation Trust Member please visit:
www.srft.nhs.uk/for-members

If you have any suggestions as to how this document could be improved in the future then please visit:
http://www.srft.nhs.uk/for-patients
